# AHL-priming functions via oxylipin and salicylic acid

**DOI:** 10.3389/fpls.2014.00784

**Published:** 2015-01-14

**Authors:** Sebastian T. Schenk, Adam Schikora

**Affiliations:** Institute for Phytopathology, Research Centre for Biosystems, Land Use and Nutrition (IFZ), Justus Liebig University GiessenGiessen, Germany

**Keywords:** AHL, quorum sensing (QS), oxylipins, SA, priming

## Abstract

Collaborative action between the host plant and associated bacteria is crucial for the establishment of an efficient interaction. In bacteria, the synchronized behavior of a population is often achieved by a density-dependent communication called quorum sensing. This behavior is based on signaling molecules, which influence bacterial gene expression. *N*-acyl homoserine lactones (AHLs) are such molecules in many Gram-negative bacteria. Moreover, some AHLs are responsible for the beneficial effect of bacteria on plants, for example the long chain *N*-3-oxo-tetradecanoyl-L-homoserine lactone (oxo-C14-HSL) can prime *Arabidopsis* and barley plants for an enhanced defense. This AHL-induced resistance phenomenon, named AHL-priming, was observed in several independent laboratories during the last two decades. Very recently, the mechanism of priming with oxo-C14-HSL was shown to depend on an oxylipin and salicylic acid (SA). SA is a key element in plant defense, it accumulates during different plant resistance responses and is the base of systemic acquired resistance. In addition, SA itself can prime plants for an enhanced resistance against pathogen attack. On the other side, oxylipins, including jasmonic acid (JA) and related metabolites, are lipid-derived signaling compounds. Especially the oxidized fatty acid derivative *cis*-OPDA, which is the precursor of JA, is a newly described player in plant defense. Unlike the antagonistic effect of SA and JA in plant–microbe interactions, the recently described pathway functions through a synergistic effect of oxylipins and SA, and is independent of the JA signaling cascade. Interestingly, the oxo-C14-HSL-induced oxylipin/SA signaling pathway induces stomata defense responses and cell wall strengthening thus prevents pathogen invasion. In this review, we summarize the findings on AHL-priming and the related signaling cascade. In addition, we discuss the potential of AHL-induced resistance in new strategies of plant protection.

## AHLs IMPACT ON PLANT PHYSIOLOGY AND DEVELOPMENT

Cell-to-cell signaling is a widespread practice in living organisms. Bacteria use a pheromone-like system called quorum sensing (QS). QS was first described in *Vibrio fischeri*, a bacterium that lives in symbiosis with a squid and produces bioluminescent light at high cell densities (Tomasz, 1965; Kempner and Hanson, 1968; Ruby and Nealson, 1976). *V. fischeri* produces *N*-acyl homoserine lactones (AHLs) that are freely diffusible across the bacterial membranes and accumulate in their surroundings (Kempner and Hanson, 1968). When a threshold concentration of AHLs is achieved, the bacterial population is able to sense (*sensing*) the critical cell density, the so-called *quorum* (Tomasz, 1965). Besides a regulation of the AHL-regulon, a very important feature of QS is the *autoinduction* of AHL-synthase expression. This communication system enables individual bacterial cells to monitor the population density and coordinate a conjoint action(s) (Waters and Bassler, 2005; Antunes and Ferreira, 2009; Teplitski et al., 2010; Nazzaro et al., 2013). In many situations, the ability of bacterial population to behave co-operatively and to communicate with each other brings clear advantages; for example, bacteria benefit from QS for conjugation, symbiotic, or pathogenic interactions with the host, for adaptation and distribution within an ecological niche (efficiency sensing; Hense et al., 2007), or for the production and secretion of secondary metabolites like antibiotics or siderophores (Williams, 2007; Hartmann et al., 2014). In Gram-negative bacteria, QS system is often based on AHLs, it is until now the best characterized bacterial communication system (Engebrecht and Silverman, 1984). AHL molecules can vary in the length of the acyl chain (4-18-carbons) and in the substitutions at the carbon chain. In addition to AHLs, 2-alkyl-4-quinolones, long-chain fatty acids, fatty acid methyl esters, and furanones (autoinducer-2) can be used for bacterial communication (Williams, 2007).

Reports from independent laboratories claimed that the short chain AHLs induce a growth promotion effect due to an impact on the phytohormone auxin (von Rad et al., 2008; Bai et al., 2012; Liu et al., 2012). The first study of AHL impact on plant hormone metabolism was performed with *Medicago truncatula* during the response to AHLs originated from the symbiotic bacterium *Ensifer meliloti* (*Sinorhizobium meliloti)*. In this study, authors revealed 150 differentially regulated proteins, within those were several auxin-induced proteins and enzymes that are involved in auxin metabolism. Furthermore, the activation of the β-*Glucuronidase* (GUS) reporter gene under the control of the auxin-responsive *GH3* promoter, indicated the involvement of auxin in the response to AHL (Mathesius et al., 2003). The possible role of auxin in response to AHL treatment was also suggested by transcriptional analyses. Auxin-associated genes were induced after a treatment with the short chain *N*-hexanoyl-homoserine lactone (C6-HSL) as well as after a pretreatment with the long chain *N*-3-oxo-tetradecanoyl-L-homoserine lactone (oxo-C14-HSL) and a subsequent challenge with the pathogen elicitor flg22 (von Rad et al., 2008; Schenk et al., 2014). In addition, genes involved in cytokinin metabolism, which have an antagonistic function to auxin, were down regulated (von Rad et al., 2008). The same report described an alteration of the free auxin to cytokinin ratio in root and shoot tissues after AHL application, explaining as a consequence the promotion of plant growth (von Rad et al., 2008). Another study showed the involvement of auxin in the AHL-induced growth as a result of the production of hydrogen peroxide and nitric oxide, which are dependent on the cyclic GMP signaling. In the postulated model, the QS molecule *N*-3-oxo-dodecanoyl-L-homoserine lactone (oxo-C10-HSL), induced an enhanced basipetal auxin transport followed by accumulation of H_2_O_2_ and NO, and stimulated therefore the formation of adventitious roots (Bai et al., 2012). Nevertheless, some publications disagree with the involvement of auxin in the AHL-growth promoting effect on plants. Despite the strong impact of oxo-C10-HSL on primary root growth and in contrast to other findings, lateral root formation, and root hair development was independent of auxin signaling as indicated by the expression analysis of the *GUS*-reporter genes under the control of the auxin-regulated *DR5* promoter (Ortiz-Castro et al., 2008). Moreover, a recent report suggested that the growth promoting effect of AHLs depends on the AHL-derivative L-homoserine, which is produced upon amidolysis of AHLs by the fatty acid amide hydrolase (Palmer et al., 2014). The authors postulated that the increased transpiration induced by L-homoserine, would enhance the water and minerals flow through plant organism and therefore positively influence the growth.

Beside the enhancement of growth, long chain AHLs have impact on plant defense mechanisms (Schikora et al., 2011; Schenk et al., 2012, 2014). In contrast to animals, plants do not have specialized cells for immune responses; for this reason, the attacked plant cell needs to reprogram its regular cellular functions for a defense response. Plants developed specialized local defense mechanisms and specific systemic responses, which are coordinated by systemic signals (Spoel and Dong, 2008). In this coordination, the cross talk between hormones plays a crucial role (Koornneef and Pieterse, 2008). The defense response against necrotrophic pathogens is usually dependent on the plant hormones jasmonic acid (JA) and ethylene (ET), while the defense reactions to biotrophic pathogens are dominantly regulated by salicylic acid (SA; Glazebrook, 2005). The antagonistic interaction between the SA and JA is well characterized (Rojo et al., 2003; Beckers and Spoel, 2006), although some reports claim a synergistic interaction between these two phytohormones (van Wees et al., 2000). An involvement of defense hormones in the AHL-induced resistance was postulated after the observation that an inoculation with the AHL-producing rhizobacterium *Serratia liquefaciens* strain MG1 enhanced systemic defense and the accumulation of SA in tomato plants (Hartmann et al., 2004; Schuhegger et al., 2006). Similar results were observed after a treatment of tomato plants with pure C6- and C4-HSL; the SA- and ET-dependent *Pathogenesis Related1a* (*PR1a*) and two *chitinase* genes were highly expressed after the treatment (Schuhegger et al., 2006). The enhanced expression of those genes in tomato leaves after application of C6-HSL or C4-HSL to the roots suggested that the systemic response functions via an SA-dependent pathway (Schuhegger et al., 2006). Likewise, an application of the long chain AHL (oxo-C14-HSL) on *Arabidopsis* roots induced a systemic response in plant shoots (Schikora et al., 2011). The AHL-induced pathway could therefore depend on SA together with the oxylipin 12-oxo-phytodienoic acid (*cis*-OPDA), as indicated by the accumulation of those two hormones, as well as mutant studies and transcriptional analyses (Schenk et al., 2014), see also chapter on AHL-priming below.

## THE SYNERGISTIC ROLE OF SA AND OXYLIPINS IN PLANT DEFENSE

Phyto-oxylipins are a diverse group of lipid-derived compounds including JA and jasmonate-related metabolites like *cis*-OPDA, methyl jasmonate, and the active form of JA, jasmonyl-*l*-isoleucine (JA-Ile). These compounds are unsaturated fatty acids produced by lipoxygenases (LOX) that oxidize the lipid chain at the C9 or C13 position (Andreou and Feussner, 2009). Additionally, oxylipins can be synthesized non-enzymatically via the free radical-catalyzed pathway, which generates similar structures denominated phytoprostanes (Sattler et al., 2006). While a lot is known about the biological function of JA, methyl jasmonate, and JA-Ile, including their perception and signal transduction (reviewed in Browse, 2009), the biological role of oxylipins before their conversion to JA is less understood. Nevertheless, several studies assumed that the precursors of JA play a role in different developing processes and during defense responses (Blee, 2002; Dave and Graham, 2012). For example, 18-cabon divinyl ether fatty acid, colneleic, and colnelenic acids accumulated in potato and tobacco leaves during the late blight disease (Weber et al., 1999). In addition, phytoprostanes accumulated as a consequence of pathogen-induced oxidative stress (ROS-production), induced the activation of Mitogen-Activated Protein Kinases (MAPKs) and glutathione-*S*-transferase (GST), expression of defense genes, and the accumulation of phytoalexin (Thoma et al., 2003). Furthermore, the enzymatically oxidized *cis*-OPDA induced expression of genes related to detoxification, stress responses, and secondary metabolism (Taki et al., 2005; Mueller et al., 2008). Interestingly, the oxylipins-related pathways induced reactions distinct from the JA-induced responses. While the expression of JA-related genes is COI1-dependent, *cis*-OPDA and phytoprostanes (PPA_1_ and PPB_1_) have been demonstrated to activate gene expression in a COI1-independent manner (Stintzi et al., 2001; Taki et al., 2005; Stotz et al., 2013). Transcriptional analysis of *Arabidopsis* showed that more than 150 genes responded to the application of *cis-*OPDA but not to JA or methyl jasmonate (Taki et al., 2005). The expression of the majority of these genes was regulated through the bZIP TGACG motif-binding transcription factors TGA2, TGA5, and TGA6 (Stotz et al., 2013). Curiously, those transcription factors are also required for the activation of SA-dependent genes (Zhang et al., 1999, 2003). A recent discovery indicated that the oxylipin pathway induced by biotic stress interacts with the SA-dependent signaling and results in a stomatal defense response (Montillet et al., 2013). The authors postulated that during stomatal defense the activation of MPK3 and MPK6 induced the guard cell lipoxygenase LOX1 and hence the peroxidation of poly unsaturated fatty acids into oxylipins followed by the accumulation of SA. Downstream of this SA accumulation was the regulation of the anion channel SLAC-1, which coordinates the stomatal defense response.

## THE PRIMING EFFECT, SENSITIZING FOR FUTURE DEFENSE RESPONSES

One of the consequences of an activated defense mechanism is a high consumption of energy. Therefore, the immune system of higher organisms needs to be coordinated in an efficient manner. In order to lower the cost of defense, plants developed different mechanisms to orchestrate their immune system, among them are negative regulators that suppress the defense response in the absence of a pathogen, or the induction of specific pathways, accordingly to the particular pathogen. Furthermore, plants may use priming as an efficient regulation of defense responses. This mechanism is based on a sensitization of the plant for a stronger and faster response. This phenomenon has been used in agriculture for plant protection since the early 1930s. Priming was usually defined as a part of induced resistance; however, the priming effect is only assessable after a subsequently challenge of the primed tissue (Conrath et al., 2002). Some priming inducers are well characterized, one of them is the non-proteinogenic amino acid β-aminobutyric acid (BABA) and another is SA at low concentrations. BABA priming functions through a SA- and abscisic acid (ABA)-dependent pathway, and induces enhanced callose depositions and tolerance to salt stress (Ton et al., 2005). In addition, BABA-induced resistance interferes with the action of the bacterial toxin coronatine (COR) from the pathogen *Pseudomonas syringae* (Tsai et al., 2011). Yet another priming inducer is the mobile metabolite azelaic acid, which induces a systemic protection via accumulation of SA (Jung et al., 2009).

Besides the accumulation of signaling components, few reports addressed the molecular mechanism of priming and explained the sensitized status of a plant. The first revealed the accumulation of the inactive form of MPK3 that can be rapidly activated upon a subsequent attack (Beckers et al., 2009). The second was the discovery of chromatin modifications on promoters of defense-related genes. In primed plants, histones in promoter regions of the defense-associated transcription factors *WRKY6, WRKY26*, and *WRKY53* are methylated (H3Kme3 and H3K4me2) and acetylated (H3K9, H4K5, and H4K12), which could explain the faster activation and the subsequent stronger stress response (Jaskiewicz et al., 2011). Interestingly, and very important for future research projects, is the fact that the primed status of a plant can be transmitted to next generations. The SA-induced defense and the resistance to the pathogen *P. syringae* were inherited to the offspring by transferring the histone methylation mechanism of relevant genes (Luna et al., 2012). Furthermore, the transgenerational priming was observed in progeny of plants treated with BABA or exposed to insect attack (Rasmann et al., 2012; Slaughter et al., 2012). However, while BABA and *P. syringe* priming are based on SA and SA-depending signaling, the insect-induced transgenerational priming is JA-dependent.

## AHL-PRIMING DEPENDS ON AN OXYLIPIN/SA-DEPENDENT PATHWAY

Considering that SA and JA precursors are crucial for long chain AHL-priming, the cross talk between SA and oxylipins seems to be an important feature of the AHL-induced resistance. Evidences that the AHL-induced priming acts via oxylipins/SA-dependent pathway are not restricted to the accumulation of phytohormones after sensitizing the plant with AHLs also genetic evidences support this dependency. Since the *Arabidopsis* mutants *coi1-16* and *jar1* behaved like wild-type plants when tested for AHL-enhanced resistance against *P. syringae*, the effect seems to be independent of the JA perception and the production of JA-Ile (Schenk et al., 2014). However, AHL-priming required Nonexpressor of PR Genes 1 (NPR1), which is the key regulator in SA-dependent defense, as indicated by the high proliferation of *P. syringae* in AHL-pretreated *npr1-1* mutant plants. The same holds true for the triple mutant *tga2/5/6*, which is impaired in the signal transduction in *cis*-OPDA- and SA-signaling cascade(s). Likewise, the AHL effect was lost in the *lox2* mutant (Schenk et al., 2014), missing the Lipoxygenase 2, one of the enzymes required for the oxidation of the unsaturated fatty acids, and hence for the oxylipin response in plants (Blee, 2002; Dave and Graham, 2012). In addition to the genetic studies, evidences that oxo-C14-HSL acts via the oxylipin/SA-induced pathway were observed at transcriptional level. For example, the enhanced expression of *GST6*, *GSTU19*, the stress responding heat shock proteins encoding genes *HSP70* and *HSP17*, and the cytochrome P450 (*CYP81D11*), which was observed after a *cis*-OPDA treatment (Mueller et al., 2008), was also visible during AHL-priming (Schenk et al., 2014). Furthermore, the independency of JA and ET during AHL-priming was strengthened by the expression patterns of prominent JA-responsive genes, *MYC2* and *VSP2,* and the ET-responsive genes *PR3, ERF5,* and *ETR1*, which were not influenced by the AHL pretreatment (Schenk et al., 2014).

## STOMATA DEFENSE RESPONSE, ONE OF THE MECHANISMS USED DURING AHL-INDUCED RESISTANCE

Stomata are openings in the epidermal layer of terrestrial plants. These pores are built up of two guard cells that regulate the opening and closure in order to establish the exchange of gasses between the leaf and the environment. This regulation system allows the control of transpiration. During drought stress, the regulation of anion-channels in guard cells is coordinated by ABA. The perception of ABA activates the guard cell-specific, ABA-related protein kinase OST1, which is followed by production of ROS and activation of Ca^2+^-signaling (Mustilli et al., 2002). Moreover, stomatal closure is tightly controlled by innate immunity as it has a crucial role during prevention of pathogen invasion. This phenomenon is referred to as stomatal defense response and functions as physical barrier against pathogen entry (Melotto et al., 2008).

A report on the inoculation of *Arabidopsis* plants with *P. syringae* showed that guard cells perceive the pathogen, indicating an active role of guard cells in plant defense (Melotto et al., 2006). However, the guard cells response to biotic stress seems to differ from the response to abiotic stress in respect to the function of the plant defense hormones SA and ABA, as well as the MAP kinases and NPR1 (Melotto et al., 2006; Zeng and He, 2010). Even though, ABA and SA signaling pathways were apparently involved in the stomatal closure induced by the beneficial bacterium *Bacillus subtilis* FB17 (Kumar et al., 2012), the above discussed report on ABA-independent pathway that controls stomatal closure in case of an immune defense response, proposed a signaling pathway, which is induced upon the perception of flg22 and includes the activation of MPK3 and MPK6 (Montillet et al., 2013). The authors observed that it requires the guard-cell-specific LOX1, producing the oxylipin *cis*-OPDA. A high accumulation of *cis*-OPDA after the flg22-elicitation in guard cells was followed by an accumulation of SA (Montillet et al., 2013).

**FIGURE 1:**
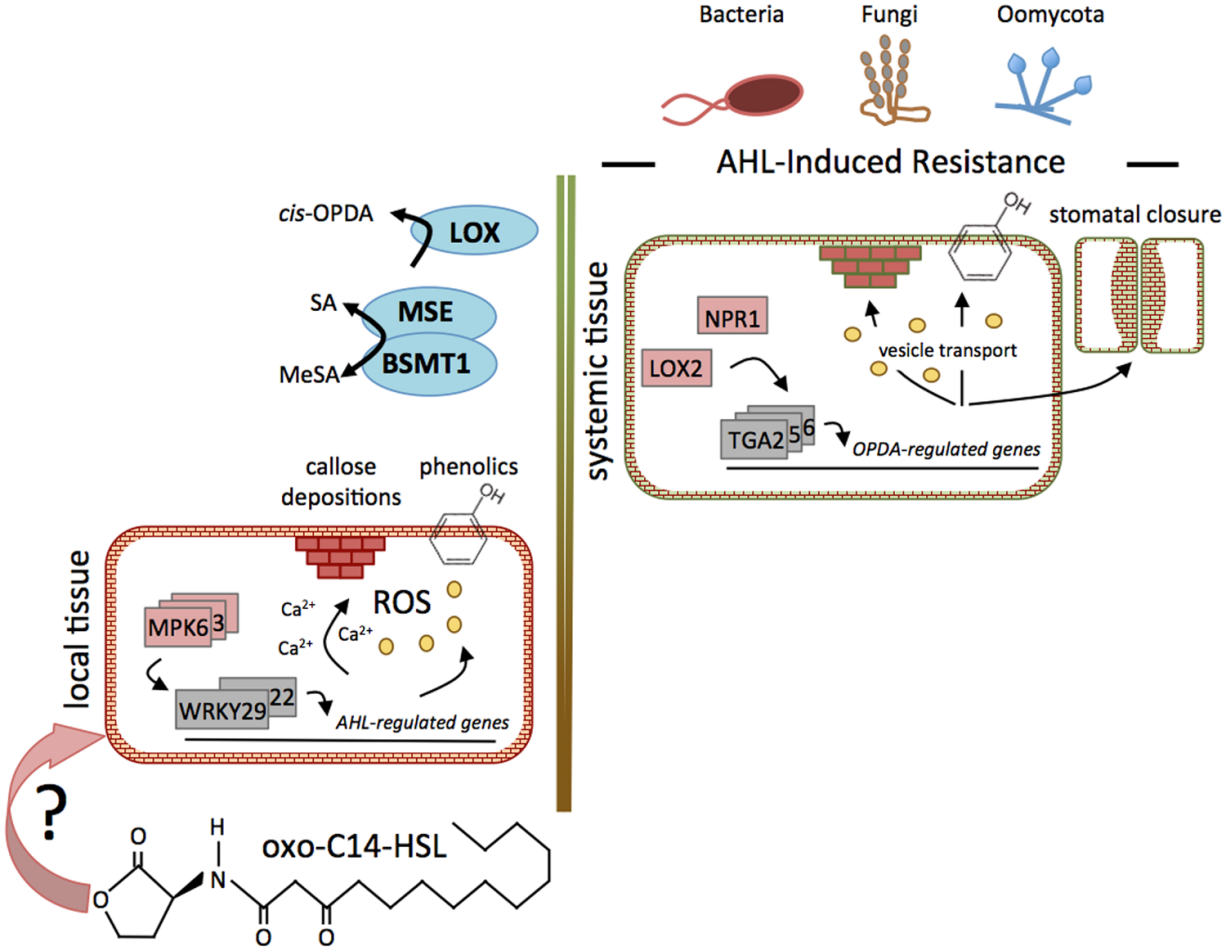
**Function of SA and oxylipins in AHL-induced priming.** Signaling steps of AHL-induced mechanisms leading to the reinforcement of resistance against several pathogens. The perception mechanism(s) of AHL in plant tissues is not known, indicated by “?”. Nonetheless, in local tissues of *Arabidopsis* plants, the oxo-C14-HSL-priming is manifested through the prolonged and stronger activation of MAPKs and the enhanced expression of *WRKY* transcription factors, followed by transcriptional reprogramming of genes related to Ca^2+^-signaling, defense, G-proteins, cell wall, and flavonoid metabolism. Furthermore, AHLs induced a higher accumulation of ROS, phenolic compounds, and callose in the cell walls. Even though long chain AHLs are not translocated to distal tissues, elevated production of the phytohormones oxylipin (*cis-*OPDA) and SA was observed in distal tissues, indicating that a systemic signaling is involved in this phenomenon. The proteins NPR1 and LOX2, as well as the TGA2/5/6 transcription factors were required. Like in the local tissue, the long chain AHL oxo-C14-HSL induced callose depositions, accumulation of phenolic compounds, and enhanced stomatal closure.

Interestingly, the AHL-induced resistance also depends on SA and *cis*-OPDA and activates the stomatal defense response (**Figure 1**). We observed an increase of closed and reduction of open stomata in oxo-C14-HSL-pretreated *Arabidopsis* plants (Schenk et al., 2014). Furthermore, the expression profile of ABA-dependent genes *RD22*, *RD29,* and *RAB18* revealed no regulation in oxo-C14-HSL-primed plants, which strengthens the postulated hypothesis on ABA-independency in stomatal defense response (Montillet et al., 2013; Schenk et al., 2014).

An important feature of phytopathogenic bacteria is the ability to reopen closed stomata, thus counteract the stomatal defense response. This is usually achieved by the bacterial toxin coronatine (COR) (Melotto et al., 2006; Zeng and He, 2010; Pieterse et al., 2014), which mimics the plant hormone JA-Ile. This virulence factor binds to the JA-receptor complex and activates the antagonistic crosstalk between SA and JA, inhibiting the flg22-triggered immune responses such as ROS production and callose depositions (Yi et al., 2014). In addition, COR suppresses the biosynthesis and accumulation of SA, hence inhibits the local and systemic defense responses (Zheng et al., 2012). Interestingly, the priming agent BABA interferes with the COR impact on stomatal defense responses. BABA-induced resistance activates the SA-dependent responses, while COR was able to suppress this defense reactions as shown by the abolished BABA effect by relatively high concentration of COR, or the failure to prime the *coi1-16* mutant (Tsai et al., 2011). Similarly, in the case of AHL-induced resistance the stomatal defense response seems to depend on SA and could disrupt the function of COR. However, the AHL-priming is still present in the *coi1-16* mutant, which indicates differences between the BABA- and AHL-priming (Schenk et al., 2014).

## AGRICULTURAL POTENTIAL OF AHL-PRIMING

Since the beginning of the twenty-first century, the bivalence between avoidance of synthetic pesticides and the performance of crop protection methods is a big challenge in agriculture. To ensure a sufficient food supply, agriculture industry has to develop modern plant protection strategies, which ensure sufficient yield and food quality. Moreover, due to market demands, farmers are under increasing pressure to produce their crops organically, or at least to reduce the chemical impact on the environment. In addition, plant production has to deal with ecological challenges like abiotic or biotic stresses and handle the arable land in the most sustainable manner. Development of new substances, which are useful in both integrated agricultural management and organic farming, is a big challenge. The development of *biologicals* or *biocontrol agents,* which originate from natural products, could be a possible strategy to meet those requirements. For instance the use of *microbial inoculants* of beneficial, soil-born microorganisms could be a competent approach to support agriculture (Berg, 2009). Using the knowledge of microbe–plant interactions, rhizosphere, or root-associated bacteria including *Bacillus*, *Pseudomonas*, and *Serratia* spp. could contribute to the production of new natural products for plant protection (Berg, 2009; Beneduzi et al., 2012; Nadeem et al., 2013). Likewise, microbial metabolites with an impact on plant growth or health have a high potential in this regard (Brader et al., 2014). The bacterial QS molecules are remarkable candidates in such strategies (Hartmann et al., 2014). Purified QS molecules and bacteria with increased production of AHLs, have an impact on plant defense mechanisms and portrait the agricultural potential of homoserine lactones (Zarkani et al., 2013; Hernández-Reyes et al., 2014). Furthermore, the use of N_2_-fixating *Rhizobia,* with their positive effects on plant physiology, could be improved by QS molecules. Nodulation efficiency, symbiosome development, exopolysaccharides production, nitrogen fixation, and adaptation to stress are all regulated by QS systems (Gonzalez and Marketon, 2003; Marketon et al., 2003). The promotion of AHL production in *Rhizobia* or *bacterial inoculants* could enhance the beneficial effects (nitrogen fixation, growth promotion, reinforced plant defense) hence, lead to a reduced use of fertilizers or conventional plant protection agents in agriculture, and in this way lower the negative impact of chemicals on the environment. Another strategy was proposed by two independent laboratories, which have bioengineered tobacco and tomato plants with different bacterial AHL-synthesis genes. These transgenic plants foster beneficial plant–bacteria interactions, and alter growth and tolerance to abiotic stress (Scott et al., 2006; Barriuso et al., 2008). However, risks and advantages of AHL-producing plants need to be assessed and require further elucidation.

## Conflict of Interest Statement

The authors declare that the research was conducted in the absence of any commercial or financial relationships that could be construed as a potential conflict of interest. The reviewer, Anton Hartmann declares that, despite having collaborated with the author Adam Schikora, the review process was handled objectively and no conflict of interest exists.

## References

[B1] Andreou A. Feussner I. (2009). Lipoxygenases – Structure and reaction mechanism. *Phytochemistry* 70 1504–1510 10.1016/j.phytochem.2009.05.008 19767040

[B2] Antunes L. C. Ferreira R. B. (2009). Intercellular communication in bacteria. *Crit. Rev. Microbiol.* 35 69–80 10.1080/10408410902733946 19514909

[B3] Bai X. Todd C. D. Desikan R. Yang Y. Hu X. (2012). N-3-Oxo-Decanoyl-L-Homoserine-Lactone activates auxin-induced adventitious root formation via hydrogen peroxide- and nitric oxide-dependent cyclic GMP signaling in mung bean. *Plant Physiol.* 158 725–736 10.1104/pp.111.185769 22138973 PMC3271762

[B4] Barriuso J. Ramos Solano B. Fray R. G. Camara M. Hartmann A. Gutierrez Manero F. J. (2008). Transgenic tomato plants alter quorum sensing in plant growth-promoting rhizobacteria. *Plant Biotechnol. J.* 6 442–452 10.1111/j.1467-7652.2008.00331.x 18384507

[B5] Beckers G. J. Jaskiewicz M. Liu Y. Underwood W. R. He S. Y. Zhang S. (2009). Mitogen-activated protein kinases 3 and 6 are required for full priming of stress responses in *Arabidopsis thaliana*. *Plant Cell* 21 944–953 10.1105/tpc.108.062158 19318610 PMC2671697

[B6] Beckers G. J. Spoel S. H. (2006). Fine-tuning plant defence signalling: salicylate versus jasmonate. *Plant Biol. (Stuttg.)* 8 1–10 10.1055/s-2005-872705 16435264

[B7] Beneduzi A. Ambrosini A. Passaglia L. M. (2012). Plant growth-promoting rhizobacteria (PGPR): their potential as antagonists and biocontrol agents. *Genet. Mol. Biol.* 35 1044–1051 10.1590/S1415-47572012000600020 23411488 PMC3571425

[B8] Berg G. (2009). Plant-microbe interactions promoting plant growth and health: perspectives for controlled use of microorganisms in agriculture. *Appl. Microbiol. Biotechnol.* 84 11–18 10.1007/s00253-009-2092-7 19568745

[B9] Blee E. (2002). Impact of phyto-oxylipins in plant defense. *Trends Plant Sci.* 7 315–322 10.1016/S1360-1385(02)02290-2 12119169

[B10] Brader G. Compant S. Mitter B. Trognitz F. Sessitsch A. (2014). Metabolic potential of endophytic bacteria. *Curr. Opin. Biotechnol.* 27 30–37 10.1016/j.copbio.2013.09.012 24863894 PMC4045207

[B11] Browse J. (2009). Jasmonate passes muster: a receptor and targets for the defense hormone. *Annu. Rev. Plant Biol.* 60 183–205 10.1146/annurev.arplant.043008.092007 19025383

[B12] Conrath U. Pieterse C. M. Mauch-Mani B. (2002). Priming in plant-pathogen interactions. *Trends Plant Sci.* 7 210–216 10.1016/S1360-1385(02)02244-6 11992826

[B13] Dave A. Graham I. A. (2012). Oxylipin signaling: a distinct role for the jasmonic acid precursor cis-(+)-12-Oxo-Phytodienoic Acid (cis-OPDA). *Front. Plant Sci.* 3:42 10.3389/fpls.2012.00042 PMC3355751 22645585

[B14] Engebrecht J. Silverman M. (1984). Identification of genes and gene products necessary for bacterial bioluminescence. *Proc. Natl. Acad. Sci. U.S.A.* 81 4154–4158 10.1073/pnas.81.13.4154 6377310 PMC345387

[B15] Glazebrook J. (2005). Contrasting mechanisms of defense against biotrophic and necrotrophic pathogens. *Annu. Rev. Phytopathol.* 43 205–227 10.1146/annurev.phyto.43.040204.135923 16078883

[B16] Gonzalez J. E. Marketon M. M. (2003). Quorum sensing in nitrogen-fixing rhizobia. *Microbiol. Mol. Biol. Rev.* 67 574–592 10.1128/MMBR.67.4.574-592.2003 14665677 PMC309046

[B17] Hartmann A. Gantner S. Schuhegger R. Steidle A. Dürr C. Schmid M. (2004). “N-acyl homoserine lactones of rhizosphere bacteria trigger systemic resistance in tomato plants,” in *Proceedings of the 11th International Congress on Molecular Plant-Microbe Interactions*, eds Lugtenberg B. Tikhonovich I. Provorov N. (St. Paul, MN: APS Press), 554–556.

[B18] Hartmann A. Rothballer M. Hense B. A. Schröder P. (2014). Bacterial quorum sensing compounds are important modulators of microbe-plant interactions. *Front. Plant Sci.* 5:131 10.3389/fpls.2014.00131 PMC3986513 24782873

[B19] Hense B. A. Kuttler C. Mueller J. Rothballer M. Hartmann A. Kreft J. U. (2007). Opinion – Does efficiency sensing unify diffusion and quorum sensing? *Nat. Rev. Microbiol.* 5 230–239 10.1038/nrmicro1600 17304251

[B20] Hernández-Reyes C. Schenk S. T. Neumann C. Kogel K. H. Schikora A. (2014). N-acyl-homoserine lactones-producing bacteria protect plants against plant and human pathogens. *Microb. Biotechnol.* 7 580–588 10.1111/1751-7915.12177 25234390 PMC4265076

[B21] Jaskiewicz M. Conrath U. Peterhansel C. (2011). Chromatin modification acts as a memory for systemic acquired resistance in the plant stress response. *EMBO Rep.* 12 50–55 10.1038/embor.2010.186 21132017 PMC3024125

[B22] Jung H. W. Tschaplinski T. J. Wang L. Glazebrook J. Greenberg J. T. (2009). Priming in systemic plant immunity. *Science* 324 89–91 10.1126/science.1170025 19342588

[B23] Kempner E. S. Hanson F. E. (1968). Aspects of light production by *Photobacterium fischeri*. *J. Bacteriol.* 95 975–979. 5643069 10.1128/jb.95.3.975-979.1968 PMC252118

[B24] Koornneef A. Pieterse C. M. (2008). Cross talk in defense signaling. *Plant Physiol.* 146 839–844 10.1104/pp.107.112029 18316638 PMC2259093

[B25] Kumar A. S. Lakshmanan V. Caplan J. L. Powell D. Czymmek K. J. Levia D. F. (2012). Rhizobacteria *Bacillus subtilis* restricts foliar pathogen entry through stomata. *Plant J.* 72 694–706 10.1111/j.1365-313X.2012.05116.x 22862801

[B26] Liu F. Bian Z. Jia Z. Zhao Q. Song S. (2012). The GCR1 and GPA1 participate in promotion of *Arabidopsis* primary root elongation induced by N-acyl-homoserine lactones, the bacterial quorum-sensing signals. *Mol. Plant Microbe Interact.* 25 677–683 10.1094/MPMI-10-11-0274 22250582

[B27] Luna E. Bruce T. J. Roberts M. R. Flors V. Ton J. (2012). Next-generation systemic acquired resistance. *Plant Physiol.* 158 844–853 10.1104/pp.111.187468 22147520 PMC3271772

[B28] Marketon M. M. Glenn S. A. Eberhard A. Gonzalez J. E. (2003). Quorum sensing controls exopolysaccharide production in *Sinorhizobium meliloti*. *J. Bacteriol.* 185 325–331 10.1128/JB.185.1.325-331.2003 12486070 PMC141839

[B29] Mathesius U. Mulders S. Gao M. Teplitski M. Caetano-Anolles G. Rolfe B. G. (2003). Extensive and specific responses of a eukaryote to bacterial quorum-sensing signals. *Proc. Natl. Acad. Sci. U.S.A.* 100 1444–1449 10.1073/pnas.262672599 12511600 PMC298792

[B30] Melotto M. Underwood W. He S. Y. (2008). Role of stomata in plant innate immunity and foliar bacterial diseases. *Annu. Rev. Phytopathol.* 46 101–122 10.1146/annurev.phyto.121107.104959 18422426 PMC2613263

[B31] Melotto M. Underwood W. Koczan J. Nomura K. He S. Y. (2006). Plant stomata function in innate immunity against bacterial invasion. *Cell* 126 969–980 10.1016/j.cell.2006.06.054 16959575

[B32] Montillet J. L. Leonhardt N. Mondy S. Tranchimand S. Rumeau D. Boudsocq M. (2013). An abscisic acid-independent oxylipin pathway controls stomatal closure and immune defense in *Arabidopsis.* *PLoS Biol.* 11:e1001513 10.1371/journal.pbio.1001513 PMC3602010 23526882

[B33] Mueller S. Hilbert B. Dueckershoff K. Roitsch T. Krischke M. Mueller M. J. (2008). General detoxification and stress responses are mediated by oxidized lipids through TGA transcription factors in *Arabidopsis*. *Plant Cell* 20 768–785 10.1105/tpc.107.054809 18334669 PMC2329937

[B34] Mustilli A. C. Merlot S. Vavasseur A. Fenzi F. Giraudat J. (2002). *Arabidopsis* OST1 protein kinase mediates the regulation of stomatal aperture by abscisic acid and acts upstream of reactive oxygen species production. *Plant Cell* 14 3089–3099 10.1105/tpc.007906 12468729 PMC151204

[B35] Nadeem S. M. Ahmad M. Zahir Z. A. Javaid A. Ashraf M. (2013). The role of mycorrhizae and plant growth promoting rhizobacteria (PGPR) in improving crop productivity under stressful environments. *Biotechnol. Adv.* 32 429–448 10.1016/j.biotechadv.2013.12.005 24380797

[B36] Nazzaro F. Fratianni F. Coppola R. (2013). Quorum sensing and phytochemicals. *Int. J. Mol. Sci.* 14 12607–12619 10.3390/ijms140612607 23774835 PMC3709803

[B37] Ortiz-Castro R. Martinez-Trujillo M. Lopez-Bucio J. (2008). N-acyl-L-homoserine lactones: a class of bacterial quorum-sensing signals alter post-embryonic root development in *Arabidopsis thaliana*. *Plant Cell Environ.* 31 1497–1509 10.1111/j.1365-3040.2008.01863.x 18657054

[B38] Palmer A. G. Senechal A. C. Mukherjee A. Ane J. M. Blackwell H. E. (2014). Plant responses to bacterial N-acyl L-homoserine lactones are dependent on enzymatic degradation to L-homoserine. *ACS Chem. Biol.* 9 1834–1845 10.1021/cb500191a 24918118 PMC4136694

[B39] Pieterse C. M. J. Zamioudis C. Berendsen R. L. Weller D. M. Van Wees S. C. M. Bakker P. A. H. M. (2014). Induced systemic resistance by beneficial microbes. *Annu. Rev. Phytopathol.* 52 347–375 10.1146/annurev-phyto-082712-102340 24906124

[B40] Rasmann S. De Vos M. Casteel C. L. Tian D. Halitschke R. Sun J. Y. (2012). Herbivory in the previous generation primes plants for enhanced insect resistance. *Plant Physiol.* 158 854–863 10.1104/pp.111.187831 22209873 PMC3271773

[B41] Rojo E. Solano R. Sanchez-Serrano J. J. (2003). Interactions between signaling compounds involved in plant defense. *J. Plant Growth Regul.* 22 82–98 10.1007/s00344-003-0027-6

[B42] Ruby E. G. Nealson K. H. (1976). Symbiotic association of *Photobacterium fischeri* with the marine luminous fish *Monocentris japonica*; a model of symbiosis based on bacterial studies. *Biol. Bull.* 151 574–586 10.2307/1540507 1016667

[B43] Sattler S. E. Mene-Saffrane L. Farmer E. E. Krischke M. Mueller M. J. Dellapenna D. (2006). Nonenzymatic lipid peroxidation reprograms gene expression and activates defense markers in *Arabidopsis* tocopherol-deficient mutants. *Plant Cell* 18 3706–3720 10.1105/tpc.106.044065 17194769 PMC1785394

[B44] Schenk S. T. Hernandez-Reyes C. Samans B. Stein E. Neumann C. Schikora M. (2014). N-acyl-homoserine lactone primes plants for cell wall reinforcement and induces resistance to bacterial pathogens via the salicylic acid/oxylipin pathway. *Plant Cell* 26 2708–2723 10.1105/tpc.114.126763 24963057 PMC4114961

[B45] Schenk S. T. Stein E. Kogel K. H. Schikora A. (2012). *Arabidopsis* growth and defense are modulated by bacterial quorum sensing molecules. *Plant Signal. Behav.* 7 178–181 10.4161/psb.18789 22307043 PMC3405712

[B46] Schikora A. Schenk S. T. Stein E. Molitor A. Zuccaro A. Kogel K. H. (2011). N-acyl-homoserine lactone confers resistance towards biotrophic and hemibiotrophic pathogens via altered activation of AtMPK6. *Plant Physiol.* 157 1407–1418 10.1104/pp.111.180604 21940998 PMC3252169

[B47] Schuhegger R. Ihring A. Gantner S. Bahnweg G. Knappe C. Vogg G. (2006). Induction of systemic resistance in tomato by N-acyl-L-homoserine lactone-producing rhizosphere bacteria. *Plant Cell Environ.* 29 909–918 10.1111/j.1365-3040.2005.01471.x 17087474

[B48] Scott R. A. Weil J. Le P. T. Williams P. Fray R. G. Von Bodman S. B. (2006). Long- and short-chain plant-produced bacterial N-acyl-homoserine lactones become components of phyllosphere, rhizosphere, and soil. *Mol. Plant Microbe Interact.* 19 227–239 10.1094/MPMI-19-0227 16570653

[B49] Slaughter A. Daniel X. Flors V. Luna E. Hohn B. Mauch-Mani B. (2012). Descendants of primed *Arabidopsis* plants exhibit resistance to biotic stress. *Plant Physiol.* 158 835–843 10.1104/pp.111.191593 22209872 PMC3271771

[B50] Spoel S. H. Dong X. (2008). Making sense of hormone crosstalk during plant immune responses. *Cell Host Microbe* 3 348–351 10.1016/j.chom.2008.05.009 18541211

[B51] Stintzi A. Weber H. Reymond P. Browse J. Farmer E. E. (2001). Plant defense in the absence of jasmonic acid: the role of cyclopentenones. *Proc. Natl. Acad. Sci. U.S.A.* 98 12837–12842 10.1073/pnas.211311098 11592974 PMC60140

[B52] Stotz H. U. Mueller S. Zoeller M. Mueller M. J. Berger S. (2013). TGA transcription factors and jasmonate-independent COI1 signalling regulate specific plant responses to reactive oxylipins. *J. Exp. Bot.* 64 963–975 10.1093/jxb/ers389 23349138 PMC3580818

[B53] Taki N. Sasaki-Sekimoto Y. Obayashi T. Kikuta A. Kobayashi K. Ainai T. (2005). 12-oxo-phytodienoic acid triggers expression of a distinct set of genes and plays a role in wound-induced gene expression in *Arabidopsis*. *Plant Physiol.* 139 1268–1283 10.1104/pp.105.067058 16258017 PMC1283764

[B54] Teplitski M. Mathesius U. Rumbaugh K. P. (2010). Perception and degradation of N-acyl homoserine lactone quorum sensing signals by mammalian and plant cells. *Chem. Rev.* 111 100–116 10.1021/cr100045m 20536120

[B55] Thoma I. Loeﬄer C. Sinha A. K. Gupta M. Krischke M. Steffan B. (2003). Cyclopentenone isoprostanes induced by reactive oxygen species trigger defense gene activation and phytoalexin accumulation in plants. *Plant J.* 34 363–375 10.1046/j.1365-313X.2003.01730.x 12713542

[B56] Tomasz A. (1965). Control of the competent state in pneumococcus by a hormone-like cell product: an example for a new type of regulatory mechanism in bacteria. *Nature* 208 155–159 10.1038/208155a0 5884251

[B57] Ton J. Jakab G. Toquin V. Flors V. Iavicoli A. Maeder M. N. (2005). Dissecting the beta-aminobutyric acid-induced priming phenomenon in *Arabidopsis*. *Plant Cell* 17 987–999 10.1105/tpc.104.029728 15722464 PMC1069713

[B58] Tsai C. H. Singh P. Chen C. W. Thomas J. Weber J. Mauch-Mani B. (2011). Priming for enhanced defence responses by specific inhibition of the *Arabidopsis* response to coronatine. *Plant J.* 65 469–479 10.1111/j.1365-313X.2010.04436.x 21265899

[B59] van Wees S. C. De Swart E. A. van Pelt J. A. van Loon L. C. Pieterse C. M. (2000). Enhancement of induced disease resistance by simultaneous activation of salicylate- and jasmonate-dependent defense pathways in *Arabidopsis thaliana*. *Proc. Natl. Acad. Sci. U.S.A.* 97 8711–8716 10.1073/pnas.130425197 10890883 PMC27013

[B60] von Rad U. Klein I. Dobrev P. I. Kottova J. Zazimalova E. Fekete A. (2008). Response of *Arabidopsis thaliana* to N-hexanoyl-DL-homoserine-lactone, a bacterial quorum sensing molecule produced in the rhizosphere. *Planta* 229 73–85 10.1007/s00425-008-0811-4 18766372

[B61] Waters C. M. Bassler B. L. (2005). Quorum sensing: cell-to-cell communication in bacteria. *Annu. Rev. Cell Dev. Biol.* 21 319–346 10.1146/annurev.cellbio.21.012704.131001 16212498

[B62] Weber H. Chételat A. Caldelari D. Farmer E. E. (1999). Divinyl ether fatty acid synthesis in late blight-diseased potato leaves. *Plant Cell* 11 485–493 10.1105/tpc.11.3.485 10072406 PMC144186

[B63] Williams P. (2007). Quorum sensing, communication and cross-kingdom signalling in the bacterial world. *Microbiology* 153 3923–3938 10.1099/mic.0.2007/012856-0 18048907

[B64] Yi S. Y. Shirasu K. Moon J. S. Lee S. G. Kwon S. Y. (2014). The activated SA and JA signaling pathways have an influence on flg22-triggered oxidative burst and callose deposition. *PLoS ONE* 9:e88951 10.1371/journal.pone.0088951 PMC3934882 24586453

[B65] Zarkani A. A. Stein E. Rohrich C. R. Schikora M. Evguenieva-Hackenberg E. Degenkolb T. (2013). Homoserine lactones influence the reaction of plants to rhizobia. *Int. J. Mol. Sci.* 14 17122–17146 10.3390/ijms140817122 23965976 PMC3759955

[B66] Zeng W. He S. Y. (2010). A prominent role of the flagellin receptor FLAGELLIN-SENSING2 in mediating stomatal response to *Pseudomonas syringae* pv tomato DC3000 in *Arabidopsis.* *Plant Physiol.* 153 1188–1198 10.1104/pp.110.157016 20457804 PMC2899927

[B67] Zhang Y. Fan W. Kinkema M. Li X. Dong X. (1999). Interaction of NPR1 with basic leucine zipper protein transcription factors that bind sequences required for salicylic acid induction of the PR-1 gene. *Proc. Natl. Acad. Sci. U.S.A.* 96 6523–6528 10.1073/pnas.96.11.6523 10339621 PMC26915

[B68] Zhang Y. L. Tessaro M. J. Lassner M. Li X. (2003). Knockout analysis of *Arabidopsis* transcription factors TGA2, TGA5, and TGA6 reveals their redundant and essential roles in systemic acquired resistance. *Plant Cell* 15 2647–2653 10.1105/tpc.014894 14576289 PMC280568

[B69] Zheng X. Y. Spivey N. W. Zeng W. Liu P. P. Fu Z. Q. Klessig D. F. (2012). Coronatine promotes *Pseudomonas syringae* virulence in plants by activating a signaling cascade that inhibits salicylic acid accumulation. *Cell Host Microbe* 11 587–596 10.1016/j.chom.2012.04.014 22704619 PMC3404825

